# SoyNet: A high-resolution Indian soybean image dataset for leaf disease classification

**DOI:** 10.1016/j.dib.2023.109447

**Published:** 2023-07-26

**Authors:** Arpan Singh Rajput, Shailja Shukla, S.S. Thakur

**Affiliations:** a Department of Electronics and Communication, Jabalpur Engineering College, Jabalpur, M.P., India; b Department of Electrical Engineering, Jabalpur Engineering College, Jabalpur, M.P., India; c Department of Mathematics, Jabalpur Engineering College, Jabalpur, M.P., India

**Keywords:** Soybean, Machine learning, Deep learning, Disease classification, Artificial intelligence

## Abstract

In order to address the challenges related to the classification and recognition of soybean disease and healthy leaf identification, it is essential to have access to high-quality images. A meticulously curated dataset named “SoyNet” has been created to provide a clean and comprehensive dataset for research purposes. The dataset comprises over 9000 high-quality soybean images, encompassing healthy and diseased leaves. These images have been captured from various angles and directly sourced from soybean agriculture fields; The soybean leaves images are organized into two sub-folders: SoyNet Raw Data and SoyNet Pre-processing Data. Within the SoyNet Raw Data folder are separate folders for healthy and diseased images captured using a digital camera. The SoyNet Pre-processing Data folder comprises resized images of 256*256 pixels and the grayscale versions of disease and healthy images, following a similar organizational structure. We captured the images using the Nikon digital camera and the Motorola mobile phone camera, utilizing different angles, lighting conditions, and backgrounds. They were taken in different lighting conditions and backgrounds at soybean cultivation fields to represent the real-world scenario accurately. The proposed dataset is valuable for testing, training, and validating soybean leaf disease classification.

**Specifications Table**

**Table:** 

Subject	Computer Science
Specific subject area	Soybean leaf Images, Deep learning, Machine learning, Artificial Intelligence, Agriculture
Type of data	Images
How the data were acquired	Images of Soybean Disease and healthy leaves were captured using a Nikon digital Camera and a Motorola mobile phone in the natural light from the cultivation area.
Data format	Raw and pre-processing data
Description of data collection	The dataset of soybean leaves images is .jpg images of 3456*4608 and 256 × 256 dimensions and resolutions are 300 dpi and 96 dpi. The soybean leaf images were captured using a Nikon digital camera and the rear camera of a Motorola mobile phone. The original images of the soybean leaves were saved in the .jpg format and had dimensions of 3456 × 4608 pixels. These images were subsequently resized to dimensions of 256 × 256 pixels. The dataset was organized into two main subfolders: preprocessing and SoyNet raw data folder. Each subfolder comprised four additional folders, namely “Camera Clicks” and “Mobile Click” for the original images, and “Healthy” and “Disease” for the categorized sub-subfolders containing the respective healthy and diseased images. Within the ”preprocessing” folder, there were grayscale and 256 × 256 preprocessed image subfolders. The images were captured under diverse backgrounds and lighting conditions. The proposed dataset is suitable for testing, training, and validating soybean leaf disease classification.
Data source location	• Institution: Jabalpur Engineering College, Jabalpur • City/Town/Region: Jabalpur, Madhya Pradesh • Country: India • Latitude and longitude for captured data: 23.1919° N, 79.9870° E Jawahar Lal Nehru Krishi Vishva-Vidyalaya Jabalpur, Madhya Pradesh. India • Latitude and longitude 23°12′36.9″N 79°56′47.7″E
Data accessibility	**Repository name**: Mendeley Data [7] SoyNet: Indian Soybean Image dataset with quality images captured from the agriculture field (healthy and disease Images) **Data identification number(doi):**10.17632/w2r855hpx8.2 **Direct URL to data:**https://data.mendeley.com/datasets/w2r855hpx8/2

## Value of the Data

• The SoyNet dataset is all-inclusive and consists of over 9000 high-quality images of two classes.
• The SoyNet dataset consists of Healthy and Disease leaves images.
• To the best of our knowledge, we present the inaugural open-access dataset of soybean leaf images, encompassing both healthy and diseased samples specific to the Madhya Pradesh region in India.
• This dataset holds significant value in the development of applications aimed at classifying and detecting soybean diseases and distinguishing healthy specimens. It serves as a valuable resource for training, testing, and validating soybean leaf classification or recognition models.
• Moreover, the dataset's utility extends to the creation of high-quality applications for soybean leaf classification, benefiting farmers, agriculture industries, researchers, and companies involved in soybean pesticide development.

## 1 Objective

The main goal of this dataset is to aid researchers in their studies by providing a wide range of soybean leaf images taken directly from real-field conditions. By capturing images from different angles and sourcing them from soybean agriculture fields, the SoyNet creates a valuable resource for those working on classifying and recognizing soybean leaf diseases.

## 2 Data Description

India, known for its strong agricultural sector, relies heavily on crop production, which serves as the backbone of its economy. Among the crops grown in the country, soybean holds a significant position, with India ranking fifth in terms of production [1]. The SOPA (Soybean Processors Association of India) confirms that Madhya Pradesh, often referred to as the “Soybean State” of India, contributes a total of 55% of the national soybean cultivation area [2,3]. However, the production of soybean faces challenges in the form of diseases and insect pests, which can cause substantial losses. Therefore, it is crucial to diagnose these issues accurately and address them promptly to safeguard soybean crops [4]. To aid in this process, a dataset has been compiled, consisting of images categorized into two sub-folders: SoyNet Raw Data and Soynet Pre-processing Data. The dataset includes images captured by a digital camera, containing folders for both healthy and diseased soybean leaves. The latter comprises images taken using a mobile phone, specifically focusing on diseased leaves. The images in the “Pre-processing SoyNet Data” folder have been resized to 256*256 pixels and converted to grayscale, mirroring the organization of the disease and healthy data. The images were clicked using a Nokia digital camera and a Motorola mobile phone. They were taken in various lighting conditions and backgrounds at soybean cultivation fields. The SoyNet dataset is proposed for utilization in testing, training, and validating soybean classification or recognition models. For further details on SoyNet dataset image management, refer to Table 1.

**Table 1:** Dataset management in SoyNet.

Soybean Image Type	Camera Click Raw Image Data	Mobile Click Raw Image Data	Camera Click Pre-processing Data	Mobile Click Pre-processing Data
Healthy Leaf				
Disease Leaf				

## 3 Experimental Design and Methods

### 3.1 Experimental Design

The Jawaharlal Nehru Krishi Vishwa Vidyalaya (JNKVV) located in Jabalpur, Madhya Pradesh, India played a crucial role in the acquisition of soybean leaf images for this research. The field at JNKVV served as the primary source for collecting samples of soybean leaves under real-field conditions. The latitude and longitude coordinates, specifically 23°12′36.9"N 79°56′47.7″E. [6].

#### 3.1.1 Description of the Soybean Crop and Management

Soybean plays a crucial role in the agricultural landscape of Madhya Pradesh, contributing significantly to its economic growth, particularly during the kharif season (June-November). The average productivity has been recorded between 796 and 885 kilograms per hectare. However, specific challenges hinder soybean production in the region, such as the limited availability of high-quality seeds of improved varieties, inadequate adoption of advanced production techniques, and the risks associated with rainfed crop cultivation.  Utilizing good quality seeds of improved soybean varieties, including JG 71, JG 315, JG 322, Pusa 391, Vishwas (Phule G 5), Vijay, Vishal, JG 218, JG 16, JG 130, JGG 1, JGK 1, and BGD 128(K).

**Fig. 1:**
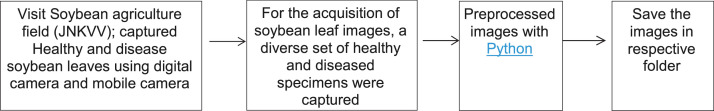
Soybean leaves data acquisition process.

The procedure for acquiring soybean leaf images is illustrated in Fig. 1. The images were obtained using two different digital cameras: the Nikon L810 and the rear camera of a Motorola G40 mobile phone. Over 9000 images were clicked using these cameras and subsequently categorized and stored in designated folders based on their quality and classification. The data acquisition procedure is outlined in Fig. 1. The Soybean leaf images were captured under natural lighting, and from different angles and backgrounds. The image pre-processing was conducted using Python. During pre-processing, the images were resized to the standard resolution of 256 × 256 pixels [5], which is optimal for building object classification.

From October to November, the data acquisition step involved capturing soybean images were captured at the stage when the soybean leaves had reached their full growth. This was done to take advantage of the natural lighting conditions and obtain a diverse range of images. The photographs were carefully taken from different angles and backgrounds, ensuring a comprehensive representation of soybean plants in their natural environment. The SoyNet dataset includes various soybean leaf diseases, including Bacterial blight, Rust, Bacterial pustule, brown spot, Downy mildew, frog leaf eye, and Soybean yellow mosaic. These disease images are stored in the respective disease folder. This meticulous approach aimed to create a dataset that accurately reflects the real-field conditions and challenges associated with soybean leaf diseases. In the subsequent phase, the pre-processing of the acquired images was conducted. Overall, the data acquisition steps outlined in Table 2 provide an overview of the timeline and activities involved in capturing and preparing the SoyNet dataset for further analysis and research.

**Table 2:** Data Acquisition steps.

S. No.	Step	Duration	Activity
1.	Data acquisition	October to November	Soybean images were captured daily, taking advantage of natural lighting conditions. The photographs were taken from various angles and backgrounds to ensure a comprehensive representation of the soybean plants in their natural environment.
2.	Pre-processing of the SoyNet dataset	December to January	Python is used to accomplish the pre-processing of the images, resize all images to a standardized resolution of 256 × 256 pixels, and then organize them into separate folders based on their classification.

Table 3 provides a comprehensive overview of common soybean leaf diseases, including their symptoms, causes, and corresponding images. It serves as a valuable reference for identifying and understanding various diseases affecting soybean crops.

**Table 3:** Description of soybean disease sample images in the SoyNet dataset.

S. No.	Disease Name	Image	Symptoms	Causes	Disease description
1	Bacterial Blight		Water-soaked lesions on leaves, Angular leaf spots, Yellowing and wilting of infected leaves	Bacterial infection	Bacterial blight is a soybean leaf disease caused by bacterial infection. It leads to water-soaked lesions, angular leaf spots, and the yellowing and wilting of infected leaves.

2	Rust		Small, reddish-brown pustules on the leaf surface, Rust-colored spores on leaves	Fungal infection	Rust is a fungal disease that affects soybean leaves. It is characterized by small, reddish-brown pustules on the leaf surface, often accompanied by rust-colored spores.
3	Brown Spot		Brown lesions with yellow halos on leaves	Fungal infection	Brown spot is a fungal disease that forms brown lesions with yellow halos on soybean leaves.

4	Downy Mildew		Yellowish-green patches on the upper leaf surface	Fungal infection	Downy mildew is a fungal disease that leads to the appearance of yellowish-green patches on the upper surface of soybean leaves.

5	Frog Leaf Eye		Small, dark green spots on leaves	Viral infection	Frog leaf eye is a viral disease characterized by the presence of small, dark green spots on soybean leaves.

6	Soybean Yellow Mosaic		Mosaic patterns on leaves	Viral infection	Soybean yellow mosaic is a viral disease that causes mosaic patterns to appear on soybean leaves.

### 3.2 Specifications of the Soybean Leaf Image Acquisition Model

The Soybean leaf pictures were obtained using two different devices: the Nikon L810, equipped with a 12-megapixel rear camera, and the Motorola, equipped with a 64-megapixel rear camera. All raw images in the dataset were originally captured at a size of 3456 × 4608 pixels. To standardize the image dimensions, python was utilized to preprocess all images to 256 × 256 pixels. The images were saved in the .jpg format.

During the image acquisition process, we visited the field and captured the leaf images directly from the plants, ensuring they were in their natural condition. Leaves were not detached from the plants for photography purposes. The methodology considered various environmental conditions, including different lighting conditions, backgrounds, and angles, to provide diverse images. The dataset was then organized into two main categories: images captured using a camera and images captured using a mobile device. These categories were divided into subfolders containing images of healthy and diseased soybean leaves, allowing for a comprehensive dataset representation.

The technical specifications of the devices employed for picture acquisition are presented in Table 4, while Table 5 outlines the specific attributes of the acquired images. In Table 6, the different classes of soybean leaf images are described, along with the corresponding number of images captured for each class.

**Table 4:** Specification of soybean leaf image acquisition device.

S. No.	Camera Particular	Details	
1	Camera Company	Nikon	Motorola
2	Camera Model	L810	Motorola G40
3	F-stop	12 MP f/2.3	64 MP, f/1.7
4	Exposure time	1/8000s	1/33 s
5	ISO Speed	ISO-1600	ISO-1120
6	Exposure bias	0 step	0 step
7	Focal length	10 mm	5 mm
8	metering mode	Pattern	Unknown
9	Flash mode	flash	No flash
10	Focal length	22.5–585 mm	25mm

**Table 5:** Specification of image.

S. No.	Particulars	Details as per soybean healthy and disease images	
		Raw images	Preprocessed images
1	Dimension	3456*4608	256*256
2	Width	3456 pixels	256 pixels
3	Height	4608pixels	256 pixels
4	Horizontal Resolution	300dpi	96dpi
5	Vertical Resolution	300dpi	96dpi
6	Bit Depth	24	24
7	Resolution unit	2	2
8	Color representation	sRGB	Grayscale

**Table 6:** SoyNet dataset details.

Quality classes	Mode of capture images	Soybean leaf classes considered	Total No. of images
Raw Images	Camera Clicks	Disease	1056
		Healthy	138
	Mobile pic	Disease	169
			

Pre-processed Images	Camera Clicks_256*256	Disease Pre-processing	3164
		Healthy Pre-processing	556
	Grayscale _Camera Clicks	Grayscale Disease	3163
		Grayscale Healthy	416
	Mobile Clicks_256*256	Disease Pre-processing	338
			
	Grayscale Mobile Click	Disease Pre-processing	338

	**Total Number of Soybean Images in the SoyNet Dataset**		**9338**

## Ethics Statements

There are no Conflicting interests involved, and the data is freely accessible in the public domain.

## CRediT authorship contribution statement

**Arpan Singh Rajput:** Methodology, Software, Validation, Conceptualization, Data curation, Visualization, Writing – original draft, Writing – review & editing. **Shailja Shukla:** Supervision, Validation. **S.S. Thakur:** Supervision, Validation.

## Data Availability

SoyNet: Indian Soybean Image dataset with quality images captured from the agriculture field (healthy and disease Images) (Original data) (Mendeley Data).
